# Multi-omics insights into uric acid metabolism in cardiometabolic disease: from genetics to metabolomics

**DOI:** 10.1016/j.bj.2026.100997

**Published:** 2026-05-25

**Authors:** Nurshad Ali

**Affiliations:** Department of Biochemistry and Molecular Biology, Shahjalal University of Science and Technology, Sylhet, 3114, Bangladesh

**Keywords:** Hyperuricemia, Serum uric acid, Multi-omics integration, Cardiometabolic disease, Precision medicine, Systems biology

## Abstract

Hyperuricemia, characterized by elevated serum uric acid (SUA) levels, is increasingly recognized as a significant contributor to cardiometabolic diseases including hypertension, type 2 diabetes, chronic kidney disease, metabolic syndrome, and atherosclerotic cardiovascular disease. In the past, uric acid was seen mainly as a byproduct of purine metabolism linked to gout. However, growing evidence suggests that it plays an active role in causing metabolic and vascular dysfunction. Mechanistic studies have shown that higher uric acid levels can induce endothelial dysfunction, oxidative stress, inflammation, insulin resistance, and activation of the renin-angiotensin-aldosterone system, which together can worsen cardiometabolic conditions. Recent advances in high-throughput omics technologies have greatly improved understanding of the molecular mechanisms regulating uric acid metabolism. Genome-wide association studies (GWAS) have identified important urate transporter genes like SLC2A9, ABCG2, and SLC22A12, while epigenomic studies reveal how DNA methylation, histone changes, and non-coding RNAs connect genetic factors to environmental influences. Transcriptomic and single-cell RNA sequencing analyses explain how urate transport and inflammatory signaling are regulated in specific tissues, including the kidneys, liver, adipose, and vascular tissue. In parallel, metabolomic and proteomic studies have linked hyperuricemia to disruptions in purine metabolism, redox balance, lipid remodeling, and inflammatory protein networks. Together, multi-omics approaches that integrate genomics, epigenomics, transcriptomics, proteomics, and metabolomics, along with expression quantitative trait locus (eQTL) mapping, causal modeling, network biology, and AI analysis, now provide powerful tools for biomarker discovery and mechanistic interpretation. This review summarizes current insights into uric acid metabolism from a multi-omics perspective and highlights emerging opportunities for better risk assessment, biomarker discovery, therapeutic targeting, and tailored prevention strategies in cardiometabolic disease.

## 1 Introduction

Hyperuricemia, defined as elevated levels of serum uric acid (SUA), is increasingly recognized as a global public health issue. Its prevalence has significantly increased in recent decades, alongside rising rates of obesity, hypertension, type 2 diabetes (T2D), and chronic kidney disease (CKD) [1,2]. Cardiometabolic diseases are the leading causes of morbidity and mortality around the world with complex metabolic, inflammatory, and vascular causes [3]. Epidemiological studies consistently show strong links between high SUA and hypertension, insulin resistance, metabolic syndrome, atherosclerosis, and CKD progression [[4], [5], [6]]. However, there has been long-standing debate about whether uric acid is merely a marker of underlying metabolic dysfunction or an active mediator in disease processes.

Historically, uric acid was mainly considered an inactive byproduct of purine metabolism, important primarily in gout. Early observational studies indicated that high SUA often occurred with cardiometabolic risk factors, but causation was unclear due to the influence of obesity, kidney dysfunction, and diet [5]. Later, experimental studies challenged the view that uric acid was merely a bystander, demonstrating that it could cause endothelial dysfunction, oxidative stress, and activate the renin-angiotensin-aldosterone system (RAAS), along with promoting the growth of vascular smooth muscle [6,7]. These results supported a potential causal role for uric acid in hypertension and metabolic disease. More recent Mendelian randomization (MR) studies have provided varied yet increasingly detailed evidence on causality, suggesting effects that depend on context and specific pathways [8].

Genetic studies have greatly improved our understanding of SUA regulation. Genome-wide association studies (GWAS) have pinpointed significant areas that affect urate transport and excretion, especially variations in genes like SLC2A9 and ABCG2, which together account for a large part of the individual differences in SUA levels [9,10]. These findings have shifted research from candidate-gene studies to large population genomics, uncovering shared genetic factors between hyperuricemia and cardiometabolic conditions. However, models that focus on single variants or genes cannot fully explain the complexity of diseases, overlapping effects, or interactions between genes and the environment.

The last ten years have seen a shift toward systems biology. Besides genomics, changes in epigenetics, transcriptomic regulation, and metabolomic changes play roles in how uric acid levels are maintained and their cardiometabolic effects. DNA methylation changes have been linked to hyperuricemia and metabolic syndrome [10]. Additionally, specific transcriptomic profiles in the kidneys and liver provide insights into how urate transporters are regulated and how inflammatory signals are activated [10]. Metabolomic studies further reveal that hyperuricemia is connected to wider disturbances in purine metabolism, oxidative stress pathways, and lipid metabolism [11]. These findings highlight the need for comprehensive studies across multiple levels.

Combining different omic data—genomics, epigenomics, transcriptomics, and metabolomics—provides a strong framework to untangle causal networks, identify molecular links, and improve risk predictions [[12], [13], [14]]. By integrating varied biological information, researchers can transition from finding associations to understanding mechanisms and then to applying that knowledge in clinical settings [14]. This approach is especially important for cardiometabolic diseases, where genetic, environmental, and metabolic factors all come together [Fig. 1].

**Fig. 1:**
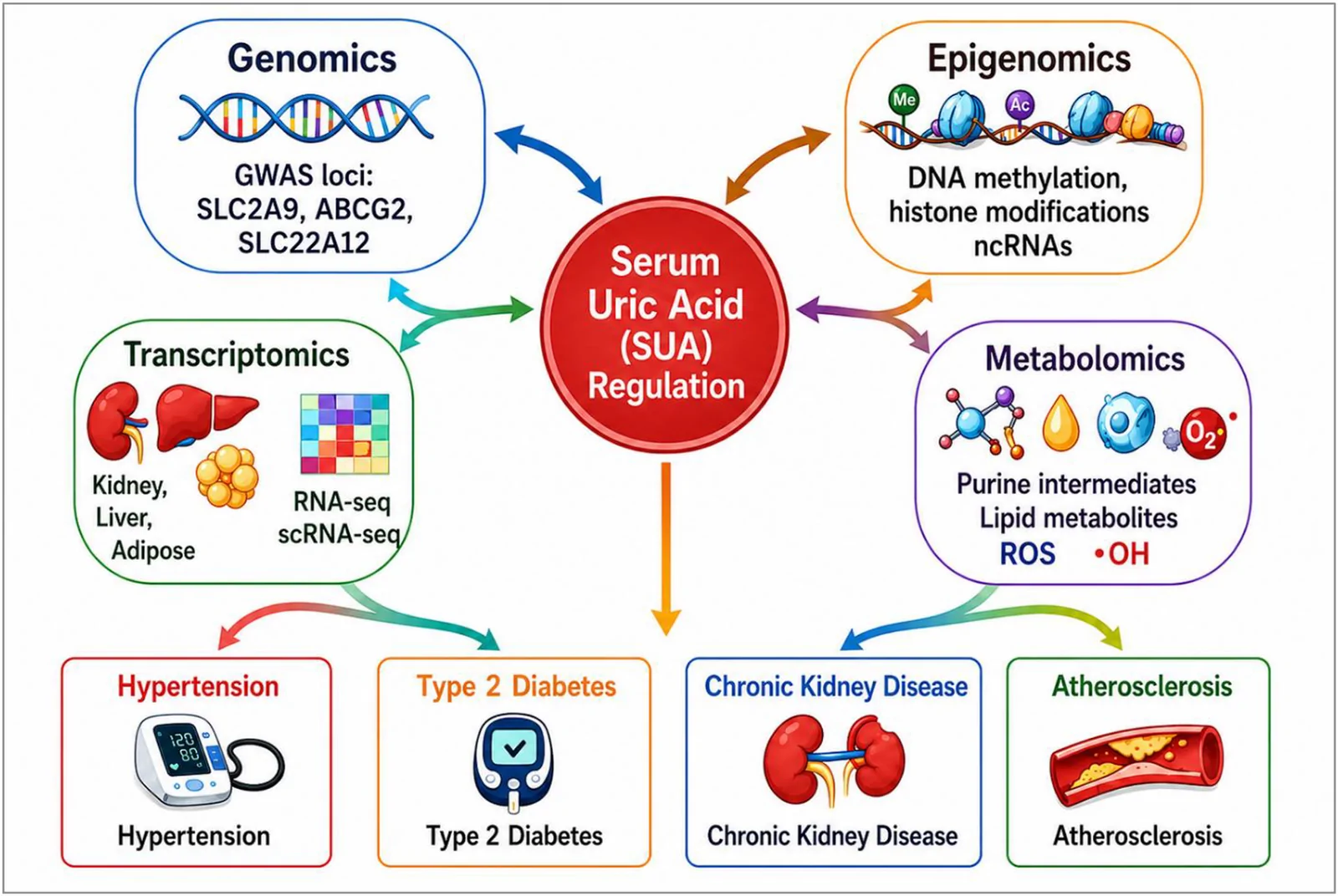
**Multi-Omics Framework of Uric Acid Metabolism in Cardiometabolic Disease.** This framework shows interactions between genomics, epigenomics, transcriptomics, and metabolomics in regulating serum uric acid. This regulation contributes to hypertension, type 2 diabetes, chronic kidney disease, and atherosclerosis. SUA, serum uric acid; GWAS, genome-wide association study; scRNA-seq, single-cell RNA sequencing; ROS, reactive oxygen species; ncRNAs, non-coding RNAs.

Recent advances in integrative analytics, such as expression quantitative trait locus (eQTL) mapping, network biology, causal inference modeling, and AI-assisted multi-omics integration, allow for more accurate characterization of molecular pathways and biomarker signatures related to hyperuricemia-associated cardiometabolic disease [15,16].

This review aims to summarize current multi-omics findings related to uric acid metabolism and its association with cardiometabolic diseases. It explores genomic, epigenomic, transcriptomic, proteomic, and metabolomic alterations associated with hyperuricemia. The review also discusses how combined analytical methods, such as eQTL mapping, network biology, causal inference modeling, and AI-assisted multi-omics integration, can help clarify cause-and-effect relationships, find important biomarkers, improve disease classification, and guide tailored prevention strategies.

A comprehensive literature search was conducted using PubMed, Scopus, Web of Science, and Google Scholar to find relevant studies published up to 2026 on uric acid metabolism, hyperuricemia, and cardiometabolic disease. Search terms included combinations of “hyperuricemia,” “serum uric acid,” “genomics,” “epigenomics,” “transcriptomics,” “proteomics,” “metabolomics,” “multi-omics,” “GWAS,” “eQTL,” “Mendelian randomization,” “cardiometabolic disease,” “chronic kidney disease,” and “precision medicine.” Prioritization was given to peer-reviewed original research articles, systematic reviews, meta-analyses, and large-scale cohort studies. Additional relevant references were identified through manual screening of the citations from selected articles.

## 2 Uric acid metabolism and cardiometabolic pathophysiology

### 2.1 Purine metabolism and uric acid production

Uric acid is the end product of purine metabolism in humans. Dietary purines, endogenous nucleotide turnover, and de novo purine synthesis converge on hypoxanthine and xanthine, and are then sequentially oxidized by xanthine oxidoreductase to produce uric acid [17]. Unlike most mammals, humans do not have uricase, which leads to higher levels of serum uric acid (SUA) and a greater risk of hyperuricemia [18]. The liver produces most of the uric acid, but endothelial cells and adipocytes also contribute during periods of metabolic stress. Increased purine breakdown, high-fructose consumption, low oxygen levels, and higher cell turnover can all raise uric acid levels by stimulating ATP depletion and xanthine oxidase activity [19].

In the body, uric acid serves as a circulating antioxidant. However, in certain conditions, it can have opposite effects, increasing oxidative stress and causing cardiovascular dysfunction [7,20]. Therefore, uncontrolled production of uric acid is closely linked to impaired metabolic and cardiovascular homeostasis.

### 2.2 Renal and intestinal urate transport mechanisms

The transporter that reabsorbs urate and is encoded by SLC22A12 (URAT1) facilitates uptake of urate from the tubular space into epithelial cells, making it important for renal urate conservation [1]. On the other hand, the transporter encoded by ABCG2 (also known as BCRP) assists in urate secretion in both the kidneys and intestines, playing a major role in eliminating urate from the body [21]. Variants of ABCG2 that do not work properly can reduce urate excretion in the intestines, which may lead to elevated uric acid levels and gout.

Other renal transporters also help with urate reabsorption and balance. Organic anion transporter 4 (OAT4; SLC22A11) and organic anion transporter 10 (OAT10; SLC22A13) are found on the apical membrane of proximal tubular cells and are involved in urate reabsorption [22]. Recent functional and genetic studies suggest that OAT10 may be a potential therapeutic treatment target, because loss-of-function variants are associated to lower serum uric acid levels and a reduced risk of gout [23,24]. These findings show that managing urate in the kidneys relies on a network of transporters, not just URAT1 and GLUT9.

### 2.3 Mechanistic links to cardiometabolic pathophysiology

Mechanistic pathways linking elevated uric acid to cardiometabolic complications are summarized below and depicted in [Fig. 2].

**Fig. 2:**
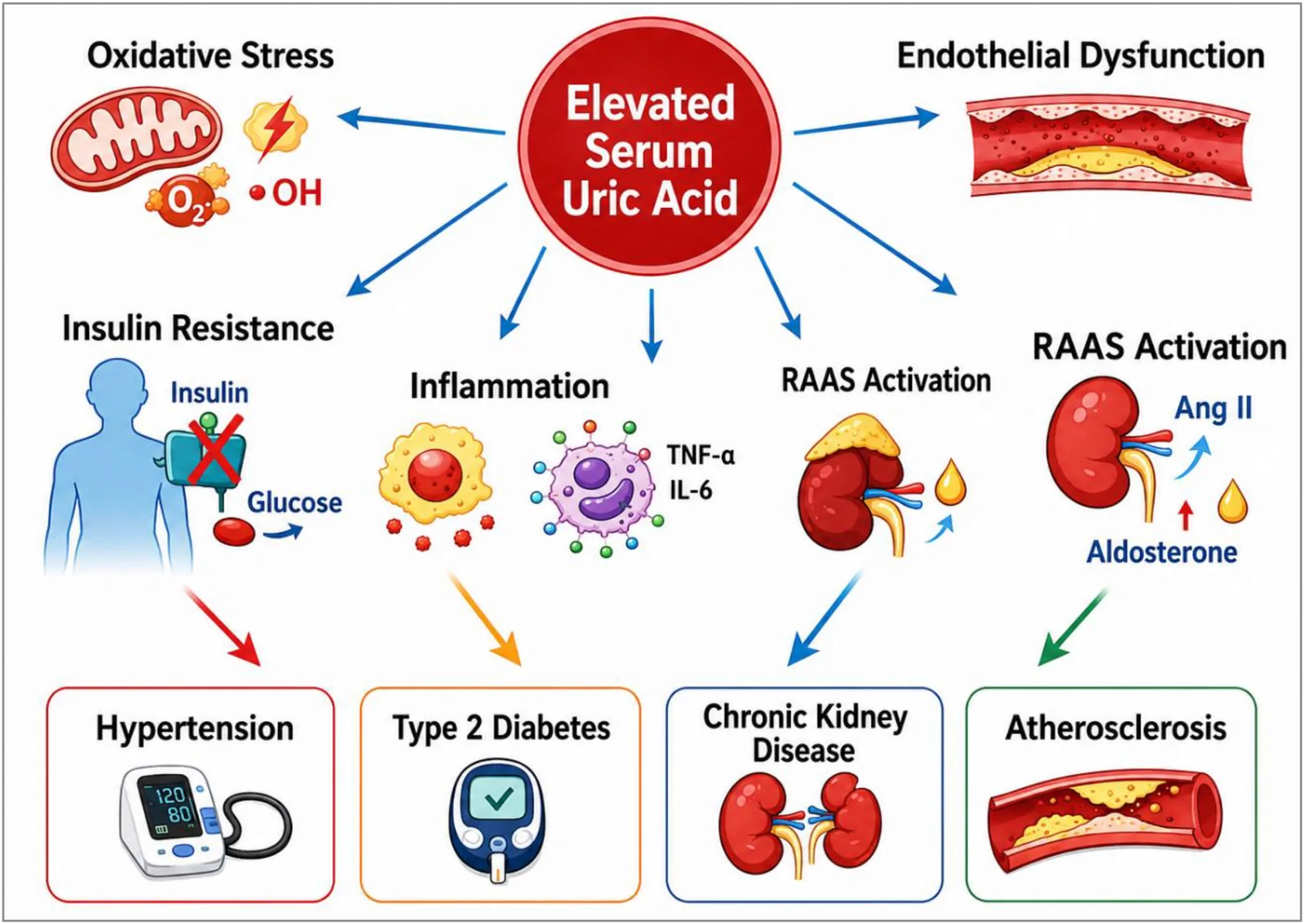
**Mechanistic Pathways Linking Serum Uric Acid to Cardiometabolic Disease.** This schematic illustrates mechanistic pathways linking high uric acid to cardiometabolic disease. Key pathways include oxidative stress, problems with blood vessel function, insulin resistance, inflammation, and RAAS activation. These factors contribute to hypertension, type 2 diabetes, and atherosclerosis. RAAS, renin–angiotensin–aldosterone system; TNF-α, tumor necrosis factor-alpha; IL-6, interleukin-6; SUA, serum uric acid.

#### 2.3.1 Endothelial dysfunction

Elevated levels of uric acid are linked to endothelial dysfunction due to lower nitric oxide (NO) availability and increased stiffness of blood vessels. Experimental studies show that uric acid blocks endothelial nitric oxide synthase (eNOS) and promotes vascular smooth muscle cell proliferation [25]. These effects impair endothelium-dependent vasodilation and lead to high blood pressure, marking uric acid as a possible cause of vascular injury [5]. In vitro and animal studies further indicate that uric acid inside cells decreases NO production by raising asymmetric dimethylarginine levels and hindering l-arginine transport [26]. Additionally, uric acid raises endothelin-1 expression, which adds to vasoconstriction and arterial stiffness [6]. Clinical studies demonstrate that elevated SUA is associated with reduced flow-mediated dilation, greater arterial stiffness, and endothelial dysfunction, highlighting its role in the progress of cardiometabolic diseases [[27], [28], [29]].

#### 2.3.2 Oxidative stress

While uric acid outside cells may act as an antioxidant, its accumulation inside cells activates NADPH oxidase and produces reactive oxygen species (ROS) in mitochondria [7]. Higher activity of xanthine oxidase further increases superoxide production, leading to lipid peroxidation, LDL oxidation, and damage to blood vessels [17]. Overproduction of ROS disrupts mitochondrial function and activates redox-sensitive transcription factors such as NF-κB, thereby promoting vascular inflammation, endothelial injury, and atherosclerotic progression linked to hyperuricemia [26,30]. Oxidative stress also reduces the ability of insulin to cause vasodilation, thereby integrating vascular and metabolic dysfunction [31].

#### 2.3.3 Insulin resistance

Elevated SUA levels are linked to insulin resistance and the onset of T2D [2,32]. Mechanistically, uric acid causes oxidative stress in fat cells and boosts levels of monocyte chemoattractant protein-1 (MCP-1), leading to macrophage infiltration in adipose tissue [33]. This inflammatory environment disrupts insulin receptor substrate-1 (IRS-1) signaling and phosphatidylinositol 3-kinase (PI3K)/Akt activation, which reduces glucose uptake in muscle. Fructose-induced ATP depletion raises uric acid levels, which then triggers hepatic de novo lipogenesis, triglyceride accumulation, and adipose fat storage, thereby promoting a vicious cycle between elevated uric acid and insulin resistance [34,35].

#### 2.3.4 Renin-angiotensin-aldosterone system activation

Uric acid activates the renin-angiotensin-aldosterone system (RAAS), which stimulates renin expression and enhances angiotensin II signaling [5]. Inducing hyperuricemia leads to thickening of afferent arterioles, glomerular hypertension, and reduce renal blood flow [6]. These effects promote sodium retention and raise systemic blood pressure. Moreover, oxidative stress from angiotensin II may further amplify uric acid-induced vascular injury, creating a cycle between high uric acid and high blood pressure [36].

#### 2.3.5 Inflammation

Elevated uric acid levels promote inflammation by activating NF-κB and increasing cytokines like IL-6 and TNF-α [6]. Monosodium urate crystals activate the NLRP3 inflammasome. However, soluble uric acid can also cause low-level inflammation without forming crystals [1].

Soluble uric acid and monosodium urate (MSU) crystals contribute to inflammation in partially different ways. Soluble uric acid mainly causes oxidative stress, endothelial dysfunction, mitochondrial damage, and activates pro-inflammatory pathways like NF-κB and MAPK, leading to chronic low-grade inflammation linked to cardiometabolic diseases [30,35,37]. On the other hand, MSU crystals provoke a stronger innate immune response by activating the NLRP3 inflammasome. This process activates caspase-1 and releases IL-1β and IL-18, which are key factors in acute gouty inflammation [38]. Understanding the effects of urate, both with and without crystals, is crucial for grasping how hyperuricemia contributes to metabolic inflammation and gout pathology.

## 3 Genomic architecture of serum uric acid

### 3.1 GWAS discoveries and major loci

GWAS have greatly improved understanding of the genetic determinants of SUA. Early large-scale meta-analyses found several loci that influence urate levels, with the strongest and most consistent associations linked to the transporter genes SLC2A9 and ABCG2 [23,39]. Variants in SLC2A9, which encodes GLUT9, account for a significant portion of SUA variability, especially in European populations. Variants in ABCG2, particularly the Q141K missense change, hinder extra-renal urate excretion and significantly increase the risk of hyperuricemia and gout [23]. Other loci include SLC22A12, SLC17A1, and GCKR, which connect urate regulation to glucose and fat metabolism [39]. Recent *trans*-ethnic GWAS, involving hundreds of thousands of people, have found even more associated loci, revealing pathways linked to renal urate transport, purine biosynthesis, inflammation and metabolic regulation [21,40]. Several loci have been identified that influence SUA levels and cardiometabolic outcomes [Table 1].

**Table 1:** Major genetic loci associated with serum uric acid and cardiometabolic traits.

Gene	Chromosome	Variant (SNP)	Effect size on SUA	Associated cardiometabolic outcomes	Population studied	Reference
SLC2A9 (GLUT9)	4p16	rs16890979	∼0.30–0.40 mg/dL increase per risk allele	Hyperuricemia, gout, hypertension	European cohorts	[39,41]
ABCG2 (BCRP)	4q22	rs2231142 (Q141K)	∼0.20–0.35 mg/dL increase	Hyperuricemia, gout, CKD risk	East Asian, European	[21,23]
SLC22A12 (URAT1)	11q13	rs893006	Moderate increase in SUA (∼0.15–0.25 mg/dL)	Hyperuricemia, gout susceptibility	European, Japanese	[39,42]
SLC17A1 (NPT1)	6p22	rs1183201	∼0.10–0.20 mg/dL decrease	Gout protection, altered renal urate transport	European populations	[39]
GCKR	2p23	rs1260326	∼0.10–0.15 mg/dL increase	Hypertriglyceridemia, metabolic syndrome	Multi-ethnic GWAS	[21]
PDZK1	1q21	rs1967017	Small increase (∼0.08–0.12 mg/dL)	Gout risk, renal urate handling	European GWAS	[39]
LRRC16A	6p22	rs742132	Small increase (∼0.07–0.10 mg/dL)	Hyperuricemia susceptibility	Japanese populations	[23]
SLC16A9	10q21	rs1171614	∼0.08–0.12 mg/dL change	Uric acid metabolism, metabolic traits	European GWAS	[39]

Abbreviations: SUA, serum uric acid; SNP, single nucleotide polymorphism; CKD, chronic kidney disease.

### 3.2 Rare variants and exome sequencing insights

While common variants explain a large part of SUA variability, rare variants with significant effects offer additional insights into the mechanisms. Whole-exome sequencing studies have revealed loss-of-function mutations in SLC2A9 and SLC22A12 linked to renal hypouricemia, emphasizing the important roles of these transporters [43]. In contrast, rare deleterious variants in ABCG2 increase the risk of gout and hinder intestinal urate excretion [23]. Both coding and non-coding variants can play a role in urate regulation and cardiometabolic risk. Coding variants are often easier to interpret because they directly alter protein sequence or function, whereas many non-coding SNPs affect regulatory mechanisms involving enhancers, chromatin accessibility, transcription factor binding, and tissue-specific gene expression [44,45]. The biological interpretation of non-coding variants is therefore challenging due to the complexity of regulatory mechanisms, not just their allele frequency [44,45]. Researchers are increasingly using integrative approaches that combine GWAS, epigenomics, eQTL mapping, and chromatin interaction analyses to functionally annotate these regulatory regions [21,44].

### 3.3 Population-specific genetic architecture

The genetic structure of SUA differs across populations. East Asian groups show stronger effects of ABCG2 variants compared to Europeans, which partly explains variations in gout prevalence and earlier disease onset [23]. In populations with African ancestry, unique allele frequencies and linkage structures affect the detectability and strength of GWAS signals [46]. Multi-ethnic meta-analyses have revealed both common and ancestry-specific genetic locations, highlight the need for diverse genomic data to avoid Eurocentric bias [21,47]. In particular, large-scale GWAS in East Asian populations, especially in Japan, have made significant contributions to identifying uric acid–associated loci and functional variants in transporter genes such as *SLC2A9* and *ABCG2* [46,48,49]. The population-specific structure also interacts with environmental factors, including diet and alcohol intake, thereby modulating cardiometabolic risk.

### 3.4 Polygenic risk scores and cardiometabolic prediction

The polygenic nature of SUA regulation has led to the development of polygenic risk scores (PRS) that combine multiple common variants. PRS for SUA show moderate ability to predict hyperuricemia and gout, and researchers are increasingly assessing their potential for predicting hypertension, chronic kidney disease, and metabolic syndrome [50]. Combining urate PRS with clinical risk factors may help better identify individuals at risk for cardiometabolic issues. However, the effectiveness of these scores varies among different ancestries due to differences in allele frequencies and effect sizes [50]. Future PRS models that include rare variants and multi-omics data may enhance their accuracy [51].

### 3.5 Mendelian randomization studies: causality vs correlation

Mendelian randomization (MR) uses genetic variants as tools to suggest causality between SUA and cardiometabolic outcomes. Several MR studies propose a causal link between genetically higher urate levels and hypertension, but the evidence for coronary artery disease T2D is weaker and inconsistent [8,52]. These differences may result from pleiotropy, mediation through kidney function, or varying biological effects depending on the context. Recent MR analyses that incorporate larger GWAS datasets and multi-variable methods try to separate direct urate effects from confounding metabolic pathways [21].

Recent studies further highlight the need for stronger causal inference frameworks to determine if SUA directly contributes to metabolic, cardiovascular, and renal diseases or mainly reflects underlying metabolic dysfunction [53,54]. Advanced MR approaches, such as multivariable MR, bidirectional MR, and phenome-wide MR, now allow for adjustments based on confounding traits like obesity, kidney function, insulin resistance, and lipid metabolism [53,55]. Integrating longitudinal cohort data with proteomics, metabolomics, and transcriptomics can help identify the molecular pathways that link urate dysregulation to tissue damage and cardiometabolic outcomes [56,57]. Additionally, single-cell omics, spatial transcriptomics, and AI-driven causal network modeling could help differentiate direct urate effects from secondary inflammatory and metabolic responses [58,59]. Future studies that include multi-ethnic cohorts, longitudinal molecular profiling, and functional experimental validation will be important for establishing causality and identifying clinically relevant targets.

Overall, genomic evidence suggests that urate-related genes contribute meaningfully to cardiometabolic traits, although the extent and specificity of causal effects remain under investigation.

## 4 Epigenetic regulation of uric acid metabolism

Epigenetic mechanisms add another layer of regulation that connects genetic susceptibility, environmental factors, and variations in SUA levels. Unlike fixed DNA sequence changes, epigenetic modifications are flexible and respond to diet, oxidative stress, inflammation, and metabolic conditions. This makes them especially relevant to cardiometabolic diseases [60]. Key epigenetic and transcriptomic regulators of urate metabolism are summarized in [Table 2].

**Table 2:** Epigenetic and transcriptomic modifiers of uric acid transport and metabolism.

Epigenetic marker/ncRNA	Target gene	Tissue	Functional consequence	Cardiometabolic relevance	Reference
DNA methylation at CpG sites near urate transporter loci	SLC2A9	Kidney (proximal tubule)	Alters transcription of GLUT9, influencing renal urate reabsorption	Hyperuricemia, hypertension, metabolic syndrome	[10]
DNA methylation signatures associated with metabolic stress	ABCG2	Kidney and intestine	Epigenetic regulation of urate secretion pathways	Gout susceptibility, cardiometabolic risk	[10,39]
Histone acetylation (H3K27ac) under oxidative stress	SLC22A12 (URAT1)	Renal proximal tubule	Increased chromatin accessibility and transporter expression	Elevated SUA, renal urate retention	[61]
miR-34a	SIRT1/oxidative stress pathways	Liver and adipose tissue	Enhances oxidative stress and metabolic dysregulation	Insulin resistance, metabolic syndrome	[62]
miR-155	Inflammatory signaling genes (NF-κB pathway)	Endothelial and immune cells	Promotes inflammatory signaling and endothelial dysfunction	Atherosclerosis, vascular inflammation	[7]
lncRNA HOTAIR	Metabolic regulatory genes	Adipose tissue	Epigenetic scaffold influencing inflammatory and metabolic gene expression	Obesity-related metabolic dysfunction	[60]
miR-143	Insulin signaling genes (IRS-1/AKT pathway)	Adipose tissue	Modulates insulin signaling and glucose metabolism	Insulin resistance, T2D	[62]
DNA methylation changes in purine metabolism genes	XDH/XOR pathway	Liver	Alters expression of enzymes involved in uric acid production	Hyperuricemia and oxidative stress	[19]

Abbreviations: ncRNA, non-coding RNA; SUA, serum uric acid; T2D, type 2 diabetes.

### 4.1 DNA methylation signatures associated with hyperuricemia

Epigenome-wide association studies (EWAS) have found different DNA methylation patterns linked to SUA levels and hyperuricemia. CpG sites near genes involved in purine metabolism, kidney transport, and inflammatory signaling show significant methylation changes correlated with circulating urate levels [10]. Changes in methylation at sites related to urate transporters can affect how genes are expressed, which may alter how the kidneys reabsorb and excrete uric acid. Additionally, methylation patterns often overlap with traits like obesity and insulin resistance, indicating shared epigenetic pathways [26]. Factors like fructose consumption and oxidative stress may further change methylation patterns, increasing the risk of hyperuricemia [19,63].

### 4.2 Histone modifications in metabolic tissues

Histone modifications, such as acetylation and methylation, control chromatin accessibility and gene expression in metabolic organs like the kidney, liver, and adipose tissue. Studies suggest that oxidative stress and inflammatory molecules can change the activity of histone acetyltransferases and deacetylases. This, in turn, affects the transcription of genes related to urate transport and inflammation [61]. In renal proximal tubule cells, histone changes might impact the expression of important urate transporters like SLC2A9 and SLC22A12, thereby influencing uric acid reabsorption [21]. While direct evidence remains limited, new chromatin profiling methods suggest that metabolic stress can lead to lasting changes in the epigenetics of renal tissue, connecting hyperuricemia to the progression of CKD [6].

### 4.3 Non-coding RNAs regulating urate transporters

Non-coding RNAs (ncRNAs), especially microRNAs (miRNAs) and long non-coding RNAs (lncRNAs), are increasingly recognized as important regulators of urate metabolism. Certain miRNAs can target transcripts involved in oxidative stress and transporter regulation, thereby affecting SUA levels [62]. Changes in miRNA expression in fat and blood vessel tissues may worsen inflammation and endothelial dysfunction in hyperuricemia [7]. LncRNAs can act as scaffolds or competing endogenous RNAs, affecting gene networks related to purine metabolism and insulin activity [60]. While the functional roles of ncRNAs in urate balance are still being explored, comprehensive transcriptomic analyses suggest coordinated regulation across metabolic tissues [21].

### 4.4 Epigenetic mediation between genetics and cardiometabolic traits

Epigenetic changes may mediate how genetic variants influence cardiometabolic traits. For instance, genetic sites linked to SUA often overlap with regulatory regions that affect methylation and gene transcription [39]. These gene-epigenome interactions might help explain the connections between hyperuricemia, high blood pressure, and insulin resistance [2]. Combining genomic and epigenomic data may clarify causal pathways and pinpoint modifiable molecular factors. Overall, epigenetic regulation plays a crucial role in linking inherited risk, environmental exposures, and cardiometabolic outcomes in uric acid metabolism.

## 5 Transcriptomic signatures in target organs

Transcriptomic studies offer important insights into gene expression patterns specific to tissues that control SUA metabolism and its effects on cardiometabolic health. Improved bulk RNA sequencing, eQTL mapping, and single-cell transcriptomics have allowed for a detailed understanding of urate transport pathways in metabolically active organs like the kidney, liver, and adipose tissue.

### 5.1 Kidney transcriptomics (proximal tubule transport regulation)

The kidney is essential for maintaining urate balance, removing about two-thirds of circulating uric acid. Transcriptomic studies show that the renal proximal tubule is the main site for urate reabsorption and secretion. Multiple transporters, such as SLC2A9, SLC22A12, and ABCG2, work together in this process [64,65]. These transporters manage the flow of urate across the membranes of proximal tubular cells. Functional experiments have confirmed that SLC2A9 is a high-capacity urate transporter found in the proximal nephron and strongly affects serum urate levels [41]. Changes in SLC2A9 or SLC22A12 can disrupt urate reabsorption, potentially leading to low or high uric acid levels in the blood [66]. Profiling renal tissues also shows that urate transport genes are co-expressed with pathways linked to oxidative stress and inflammation. This suggests that metabolic stress in the kidney may impact how urate is managed [64].

### 5.2 Hepatic and adipose tissue expression profiles

In addition to kidney function, the liver and adipose tissue play roles in urate metabolism through purine synthesis, fructose metabolism, and inflammatory signaling. Hepatic transcriptomic studies show that enzymes involved in breaking down purines and xanthine oxidoreductase activity influence systemic urate production [17]. In adipose tissue, changes in gene expression related to oxidative stress and inflammation are tied to higher SUA levels and metabolic syndrome [67]. Further transcriptomic profiles of adipocytes reveal connections between urate metabolism and insulin signaling pathways, suggesting that high uric acid levels could contribute to metabolic dysfunction [2]. Overall, these findings indicate that urate metabolism links with metabolic regulation across different organs.

### 5.3 Integrative transcriptomics: eQTL and colocalization analyses

Instead of being an independent omics layer, eQTL analysis is an analytical framework that combines genomic variation and transcriptomic regulation to identify how disease-related variants affect gene expression in specific tissues [68,69].

eQTL analyses provide a robust framework for linking genetic variants found in GWAS to tissue-specific changes in gene expression. Many urate-related loci show strong eQTL effects in kidney tissues, especially among transporter genes like SLC2A9 and ABCG2 [39]. Colocalization analyses that combine GWAS and transcriptomic data suggest that regulatory variants affect SUA levels by changing the expression of transporter genes in kidney and intestinal tissues. These combined approaches enhance our understanding of how genetic signals relate to the mechanisms that control urate levels.

### 5.4 Single-cell RNA sequencing insights

Recent developments in single-cell RNA sequencing (scRNA-seq) have allowed for precise mapping of urate-related gene expression in different renal cell types. These datasets show that proximal tubular epithelial cells have the highest expression of urate transporters, confirming their primary role in urate reabsorption and secretion [70]. The analyses also uncover new cellular diversity within nephron segments and highlight the interactions between epithelial, immune, and supportive cells when faced with increased urate levels. Merging single-cell transcriptomics with organoid models and spatial transcriptomic techniques further improves our understanding of how genetic variation affects urate transport and renal injury pathways [71,72].

## 6 Metabolomics and systems-level metabolic remodeling

Metabolomics provides a systems-level view of biochemical activity and connects genetic factors, environmental exposures, and cardiometabolic traits. In cases of hyperuricemia, metabolomic research has shown significant metabolic changes involving purine turnover, oxidative stress pathways, and lipid metabolism. These changes help clarify how SUA contributes to broader cardiometabolic issues rather than just serving as a marker of purine metabolism [6].

### 6.1 Purine pathway intermediates

The purine metabolic pathway is the most direct source of uric acid production. Metabolomic profiling often finds increased levels of upstream intermediates like inosine, hypoxanthine, and xanthine in people with hyperuricemia. These metabolites indicate higher purine turnover or lower clearance and are closely linked to xanthine oxidoreductase activity, the enzyme that converts xanthine to uric acid [17]. Large studies in metabolomics have shown that changes in purine intermediates strongly relate to SUA levels and gout risk, suggesting that these metabolite patterns might be early signs of disrupted purine balance [73].

### 6.2 Oxidative stress and redox imbalance markers

Hyperuricemia is connected to markers of oxidative stress and redox imbalance. While uric acid can act as an antioxidant in extracellular areas, excess urate in cells increases oxidative stress by activating NADPH oxidase and causing mitochondrial dysfunction. Metabolomic studies often find altered levels of glutathione, cysteine, and other redox-related metabolites in individuals with higher SUA [6]. These changes indicate a shift in cellular antioxidant defenses and provide biochemical evidence linking uric acid metabolism with oxidative stress pathways related to cardiometabolic disease [67].

### 6.3 Lipidomic alterations in hyperuricemia

Recent lipidomic studies show significant changes in lipid metabolism related to hyperuricemia. Higher SUA levels are linked to increased triglycerides, changes in phospholipid types, and alterations in fatty acid metabolism, suggesting interactions between purine and lipid pathways. Population-based metabolomic studies have indicated that uric acid positively correlates with metabolites involved in fat production and fatty acid breakdown, highlighting its connection with obesity, insulin resistance, and metabolic syndrome [74,75].

### 6.4 Proteomics and protein network alterations in hyperuricemia

Recent proteomic studies have improved understanding of cardiometabolic dysfunction through identifying protein networks involved in oxidative stress, inflammation, endothelial injury, and lipid metabolism. High-throughput analyses of plasma proteins showed that circulating proteins tied to inflammation, cell adhesion, and metabolic control are strongly linked to cardiometabolic traits and urate-related pathways [76]. In endothelial cells, global proteomic profiling revealed that exposure to uric acid changes redox-sensitive proteins, chaperones, and inflammatory factors, leading to oxidative stress, protein misfolding, and endothelial dysfunction [77]. Combined proteomic and metabolomic approaches further showed that disruptions in lipid metabolism, immune responses, and mitochondrial functions occur in hyperuricemia and cardiovascular dysfunction [78,79]. These findings suggest that proteomics can enhance metabolomics by offering functional insights into the molecular pathways connecting high serum uric acid levels with cardiometabolic disease.

### 6.5 Integrated metabolite networks predicting cardiometabolic risk

Systems-level metabolomic studies increasingly combine multiple metabolic pathways to predict cardiometabolic risk. Network models show that uric acid is part of a wider metabolic module that includes amino acid metabolism, lipid turnover, and oxidative stress pathways. These integrated metabolite networks can improve predictions of cardiometabolic outcomes beyond traditional clinical indicators [80]. These findings emphasize the role of metabolomics in uncovering the systemic metabolic effects of hyperuricemia and highlight the value of integrating various omics for understanding the mechanisms of cardiometabolic disease [81].

## 7 Cross-omics integration and network biology

### 7.1 Multi-layer omics integration frameworks

Researchers have developed several computational frameworks to combine different omics datasets. Techniques like multi-omics factor analysis, Bayesian integration models, and network-based methods uncover shared biological signals across datasets. Emerging multi-omics studies that combine proteomics, metabolomics, and transcriptomics improve the identification of inflammatory, oxidative stress, and endothelial signaling pathways associated with hyperuricemia and cardiometabolic disease progression [63,76]. These frameworks link genomic variants associated with SUA levels to changes in transcription and metabolism in relevant tissues. Integrative analyses show that mixing genomic and metabolomic data leads to a better identification of pathways that affect cardiometabolic traits than using individual datasets [12,73,82].

Recent advances further expand the multi-omics framework by including chromatin accessibility profiling with ATAC-seq and three-dimensional chromatin interaction mapping using Hi-C technologies [83,84]. ATAC-seq helps identify regulatory elements and open chromatin regions that control urate transporter genes. Hi-C connects distal regulatory regions to target gene expression, linking genomic variation to transcriptomic regulation [83,84]. Additionally, proteomics plays a vital role between transcriptomics and metabolomics by profiling protein abundance, post-translational modifications, inflammatory mediators, and metabolic enzymes involved in urate metabolism and cardiometabolic dysfunction [85,86]. Together, integrating genomics, epigenomics, chromatin interaction mapping, transcriptomics, proteomics, and metabolomics provides a more complete framework for understanding hyperuricemia and its cardiometabolic consequences [83,86]. Several multi-omics studies integrating genomics, transcriptomics, and metabolomics are summarized in Table 3, highlighting clinical relevance and limitations.

**Table 3:** Integrative multi-omics frameworks and analytical strategies in uric acid–related cardiometabolic disease.

Country, Year	Cohort size	Primary omics layers	Integrative strategy	Biological insight	Translational relevance	Reference
UK/International, 2019	∼457,000 (UK Biobank and global consortia)	Genomics, transcriptomics, metabolomics	GWAS integration, colocalization analysis, Mendelian randomization	Identified multiple urate-associated loci linked to renal urate transport, metabolic regulation, and inflammatory pathways	Improved identification of genetic drivers of hyperuricemia and potential therapeutic targets; limited by predominantly European ancestry	[21]
Iceland/Europe, 2013	∼140,000 multi-cohort meta-analysis	Genomics, transcriptomics	GWAS meta-analysis with eQTL integration	Discovered 18 novel loci influencing SUA regulation and kidney transport mechanisms	Provided functional interpretation of GWAS loci through tissue-specific gene regulation; limited functional validation	[39]
Germany/Europe, 2011	∼2800 (KORA cohort)	Metabolomics, genomics	Network-based metabolite–gene integration	Linked uric acid metabolism with purine intermediates, lipid remodeling, and oxidative stress pathways	Identified metabolite networks predictive of cardiometabolic risk; modest cohort size limited generalizability	[73]
USA, 2012	108 individuals (longitudinal profiling)	Genomics, transcriptomics, proteomics, metabolomics	Longitudinal multi-omics profiling with network modeling	Revealed dynamic molecular and metabolic changes associated with insulin resistance and metabolic dysregulation	Demonstrated feasibility of individualized multi-omics monitoring; limited by small sample size	[87]
USA/Europe, 2016	∼7000 (multi-cohort MR analysis)	Genomics, metabolomics	Mendelian randomization and causal inference modeling	Supported potential causal relationships between elevated SUA and hypertension-related pathways	Strengthened causal inference for urate-related cardiometabolic risk; pleiotropy remained a limitation	[8]
Finland/Europe, 2016	∼10,000 population cohorts	Metabolomics, genomics	Integrated metabolite network analysis	Identified metabolite signatures associated with urate metabolism, oxidative stress, and cardiometabolic disease progression	Provided candidate biomarkers for cardiometabolic risk prediction; metabolite coverage remained incomplete	[80]
USA/Global, 2018	Multiple population cohorts (>50,000 combined datasets)	Genomics, transcriptomics, metabolomics	Machine learning, systems biology, and multi-omics integration frameworks	Demonstrated that integrated multi-omics approaches improve disease classification and molecular pathway discovery	Highlighted the clinical potential of AI-assisted precision medicine; real-world implementation still limited	[58]
China/International, 2026	Multi-cohort AI-integrated datasets	Genomics, transcriptomics, proteomics, metabolomics	Deep learning, graph neural networks, explainable AI	Identified hidden molecular interactions linking urate dysregulation with inflammation, endothelial dysfunction, and lipid remodeling	Supports precision classification and individualized therapeutic targeting in cardiometabolic disease; requires external validation	[16]

The table summarizes important studies that combine genomics, transcriptomics, proteomics, metabolomics, and computer modeling to elucidate molecular mechanisms, causal pathways, and practical applications that connect hyperuricemia with cardiometabolic disorders. **Abbreviations:** SUA, serum uric acid; eQTL, expression quantitative trait locus; GWAS, genome-wide association study; MR, Mendelian randomization; AI, artificial intelligence.

### 7.2 Systems biology and pathway enrichment

Systems biology uses pathway enrichment and network analysis to find functional modules related to disease traits. In hyperuricemia research, pathway analyses often point out biological processes related to purine metabolism, kidney transport systems, oxidative stress, and inflammatory signaling. These analyses help place molecular findings into broader biological pathways and show how uric acid metabolism interacts with cardiometabolic regulatory networks [6,67].

### 7.3 Causal network modeling

Causal network modeling adds insight by distinguishing between correlation and potential causal relationships among molecular features. Methods like MR and Bayesian network modeling connect genetic instruments with molecular traits to infer pathways that link uric acid metabolism with cardiometabolic outcomes. These methods suggest that genetic increases in SUA may affect hypertension, insulin resistance, and kidney dysfunction through linked metabolic and inflammatory pathways [39,80].

### 7.4 AI and machine learning for biomarker discovery and precision classification

Multi-omics biomarker discovery requires careful prioritization strategies to separate biologically meaningful markers from background noise. Recent studies increasingly combine statistical significance, pathway enrichment, network centrality, reproducibility across cohorts, and causal inference methods to find clinically relevant biomarkers linked to hyperuricemia and the progression of cardiometabolic disease [15,58,88].

Integrative biomarker frameworks often focus on molecules that show concordance across omics such as genomic variants linked to transcriptomic changes and related metabolomic or proteomic alterations. Such consistency across layers improves biological understanding and predictive strength compared to using single-omics biomarkers alone [16,58].

AI and machine learning methods are increasingly used to classify cardiometabolic phenotypes. Deep learning, graph neural networks, and explainable AI models can combine genomic, transcriptomic, proteomic, metabolomic, and clinical data to identify molecular subtypes and predict disease developments [16,59]. Recent studies show that AI-assisted multi-omics integration enhances the classification of metabolic syndrome, CKD progression, and cardiovascular risk compared to traditional clinical models [16,88,89].

Network-based AI frameworks may also reveal hidden molecular interactions linking urate dysregulation to core metabolic pathways associated with hyperuricemia. These methods provide opportunities for precision prevention strategies and tailored treatment in hyperuricemia-related cardiometabolic disease [16,59].

## 8 Clinical translation and precision medicine

### 8.1 Multi-omics biomarkers for risk stratification

Multi-omics methods allow for the identification of biomarker panels that better reflect the biological processes behind disease risk. In the case of hyperuricemia, genomic variants in urate transporter genes, along with transcriptomic and metabolomic signatures, can enhance the prediction of cardiometabolic outcomes beyond SUA levels alone [39,73]. Integrative analyses have also pointed out metabolite signatures linked to purine metabolism, oxidative stress, and lipid pathways related to hypertension, diabetes, and CKD [67].

### 8.2 Drug target identification and repurposing

Multi-omics data can highlight molecular pathways that may be suitable for treatment. Genetic and transcriptomic studies have found key urate transporters—including SLC2A9, SLC22A12, and ABCG2—that regulate uric acid levels and could serve as drug targets [41,42]. Current urate-lowering treatments, such as xanthine oxidase inhibitors like allopurinol and febuxostat, act early in purine metabolism. Emerging strategies aim to adjust renal and intestinal urate transport pathways [90]. Integrative omics analyses may also help repurpose drugs by identifying shared molecular pathways between hyperuricemia and cardiometabolic disorders [91].

### 8.3 Gene–environment and diet interactions

The onset of hyperuricemia is strongly affected by environmental factors and dietary habits interacting with genetic predispositions. High fructose intake, alcohol consumption, and diets rich in purines can raise uric acid production and increase cardiometabolic risk, especially in individuals with genetic variants that influence urate transport or metabolism [67,92]. Multi-omics methods allow for the study of these gene–environment interactions by linking dietary patterns to changes in epigenetic marks, gene expression, and metabolic pathways.

### 8.4 Implications for personalized cardiometabolic prevention

In the end, combining multi-omics data into clinical practice may enable tailored prevention strategies for cardiometabolic diseases. PRS, along with metabolomic and transcriptomic biomarkers, could help identify those at high risk of complications from hyperuricemia and guide early lifestyle or drug interventions [58,93]. These personalized approaches may enhance disease prevention by adjusting interventions according to an individual's molecular risk profile [93]. A pipeline for integrating multi-omics data to identify biomarkers and inform precision medicine is shown in [Fig. 3].

**Fig. 3:**
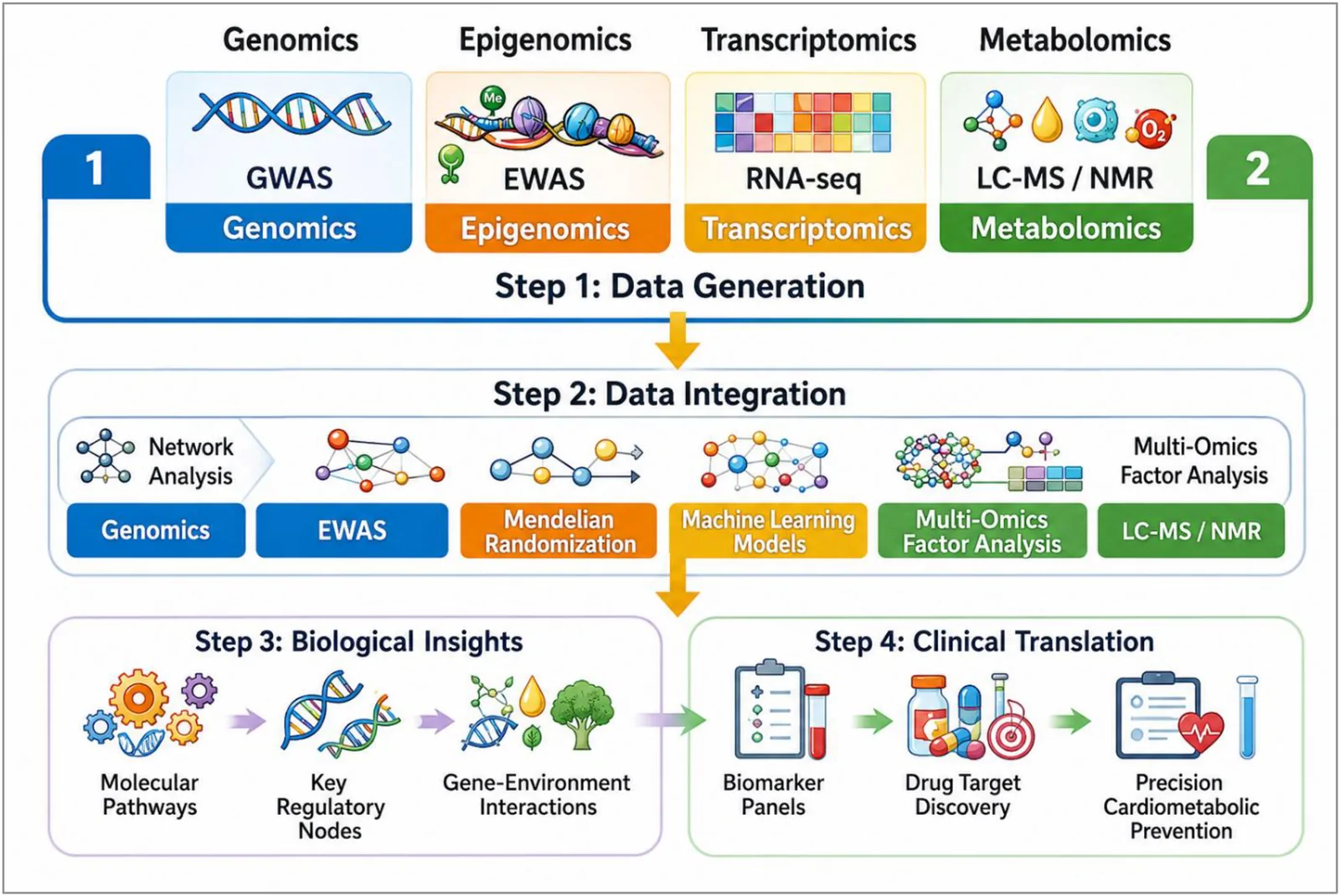
**Multi-Omics Integration Pipeline for Biomarker Discovery and Precision Medicine.** This figure shows the workflow from multi-omics data generation to biomarker discovery and clinical translation. GWAS, genome-wide association study; EWAS, epigenome-wide association study; RNA-seq, RNA sequencing; LC-MS, liquid chromatography–mass spectrometry; NMR, nuclear magnetic resonance; ML, machine learning.

## 9 Current challenges and future directions

Despite significant progress in multi-omics research on uric acid metabolism, several challenges remain before we can turn these findings into strong clinical applications. One major limitation is the lack of ethnic diversity in genomic and multi-omics datasets. Most GWAS and integrative omics analyses have mainly focused on European populations [21,39,47]. PRS primarily based on European groups have less predictive accuracy in non-European populations, such as African and South Asian groups, due to differences in allele frequencies and the structure of linkage disequilibrium. [94,95]. Notably, several large-scale GWAS conducted in Japanese and broader East Asian groups have provided significant insights into uric acid genetics, particularly showing stronger effects of ABCG2 variants and population-specific loci linked to urate excretion [46,48,49]. Variants in urate transporters like SLC2A9 and ABCG2 display differences in allele frequencies and effect sizes across populations. These differences influence the risk of hyperuricemia and gout [39,48]. Therefore, it is essential to expand multi-omics studies to include African, South Asian, and underrepresented East Asian subpopulations to achieve more equitable and globally representative precision medicine frameworks [12,96].

Another key challenge is the standardization of multi-omics analytical methods. Variations in sample preparation, sequencing platforms, data normalization, and statistical methods can cause differences in results across studies. We need to harmonize protocols and develop standardized bioinformatic methods to ensure consistent and comparable results in cross-cohort analyses [12,58]. Large-scale collaborative projects and shared data repositories will play a crucial role in supporting this standardization and enabling meta-analyses across varied datasets.

Integration of longitudinal cohort data is also limited. Most multi-omics studies on hyperuricemia and cardiometabolic disease depend on cross-sectional data, which restricts our ability to understand causal relationships and the timing of disease progression. Longitudinal multi-omics profiling could show dynamic molecular changes that occur before cardiometabolic complications. Proteomic profiling and causal mediation analyses may further help identify downstream effector pathways and distinguish direct urate toxicity from secondary metabolic effects [57]. This understanding could lead to earlier risk prediction and preventive measures [67,87]. Combining repeated omics measurements with detailed clinical data may help identify early molecular signs that connect uric acid dysregulation to metabolic and cardiovascular outcomes.

Finally, advances in AI and machine learning provide exciting opportunities to uncover complex causal relationships within high-dimensional multi-omics data. Network-based AI approaches can combine genomic, epigenomic, transcriptomic, and metabolomic data to find key regulatory points and predictive biomarkers. These methods may help clarify causal pathways linking uric acid metabolism to cardiometabolic disorders and guide the discovery of new therapeutic targets [59,97].

Future research should prioritize diverse population cohorts, standardized analysis methods, longitudinal multi-omics datasets, and AI-driven causal modeling [98,99]. Together, these strategies will improve our understanding of uric acid biology and speed up the application of multi-omics insights to precision cardiometabolic. Medicine.

## 10 Conclusion

Uric acid metabolism is increasingly seen as a key factor in cardiometabolic disease rather than just a marker of gout. Evidence from genomics, epigenomics, transcriptomics, proteomics, and metabolomics shows that elevated serum uric acid contributes to endothelial dysfunction, oxidative stress, inflammation, insulin resistance, and kidneys and vascular injury. Integrating data from multiple fields has greatly improved understanding of the molecular systems involved in cardiometabolic disorders linked to hyperuricemia and has found potential biomarkers and treatment targets. Emerging analytical frameworks like eQTL mapping, causal inference, network biology, and AI-based data integration, further enhance the ability to characterize disease mechanisms and improve risk predictions. However, significant challenges still exist, including limited diversity in study populations, lack of long-term data, and differences in methods used across studies. Future research should focus on including diverse populations, standardizing analytical methods, and improving computational models to facilitate translation into precision cardiometabolic medicine.

## Funding

This study did not receive any funding

## Declaration of competing interest

None.
