# Supplementary material for: Multi-omics insights into uric acid metabolism in cardiometabolic disease: from genetics to metabolomics
Source: Biomed J. 2026 May 25;49(5):100997. doi: 10.1016/j.bj.2026.100997 (PMC13634095; doi:10.1016/j.bj.2026.100997)
Supplement: Supplementary file 1 [file mmc1.pdf]

CURRICULUM VITAE OF DR. MD. NURSHAD ALI

**Personal Information**<br>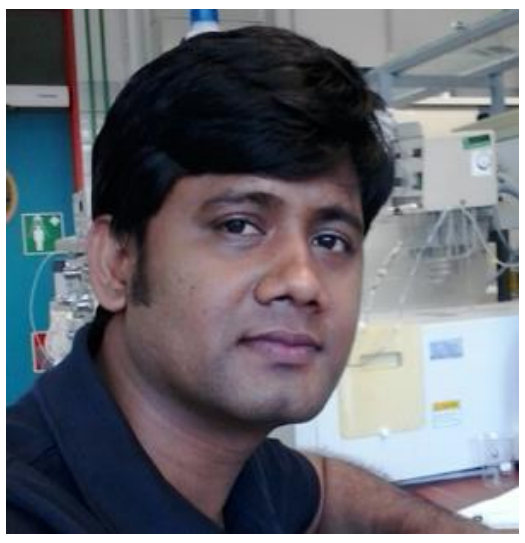	**Name: Md Nurshad Ali**<br>Dept. of Department of Biochemistry and Molecular Biology, Shahjalal University of Science and Technology, Sylhet-3114, Bangladesh<br>Permanent address**:** Village and Post Office: Mongol Para, Police Station: Puthia, District: Rajshahi, Postcode: 6251, Bangladesh<br>nali-bmb@sust.edu; nur_rubd@yahoo.com<br>Date of Birth: 15.10.1982<br>+880 1719840498. Nationality: Bangladeshi. Sex: Male, Date of Birth: 15.10.1982<br>Website: http://www.sust.edu/d/bmb/faculty-profile-detail/574/10<br>ORCID: http://orcid.org/0000-0003-1649-0887
**Professional Experience**<br>02/2022-to present	Associate Professor<br>Department of Biochemistry and Molecular Biology<br>Shahjalal University of Science and Technology, Sylhet-3114, Bangladesh
01/2018-1/2022	Assistant Professor<br>Department of Biochemistry and Molecular Biology<br>Shahjalal University of Science and Technology, Sylhet-3114, Bangladesh
01/2017-12/2017	Lecturer<br>Department. of Biochemistry and Molecular Biology<br>Shahjalal University of Science and Technology, Sylhet-3114, Bangladesh
05/2010-12/2016<br>(06/2012-03/2016 study leave for PhD.)	Lecturer<br>Department Biochemistry and Molecular Biology<br>Gono University, Savar, Dhaka1344, Bangladesh
**Education and Training**<br>11/2021-06/2024	Postdoctoral Researcher, Imperial College London, and Queen Mary University of London, UK.<br>Visiting Researcher: University College London, UK
09/2012–3/2016<br>(06/2012–09/2012 German language courses (A1 and A2), InterDaF, Leipzig, Germany)	Ph.D. in Natural Sciences<br>Leibniz Research Center for Working Environment and Human Factors<br>TU Dortmund University, Dortmund, Germany<br>Dissertation title: Biological monitoring and exposure assessment for-mycotoxins in inhabitants of Bangladesh and Germany.<br>Supervisor: Prof. Gisela H. Degen
01/2006–12/2006<br>(Due to academic session jam exam was held in 01/2009)	Master of Science (M.Sc.) in Biochemistry and Molecular Biology<br>Results: First class (Top grade A)<br>University of Rajshahi, Bangladesh<br>Thesis title: Plasma Cholinesterase activity as a biomarker for arsenicosis in humans.<br>Supervisor: Prof. Khaled Hossain
01/2002–12/2005<br>(Due to academic session jam exam was held in 01/2007)	Bachelor of Science (B.Sc.) in Biochemistry and Molecular Biology<br>Results: First class (Top grade A)<br>University of Rajshahi, Bangladesh<br>Internship: In-plant training in a pharmaceutical company
**Prizes and Awards**	i) Gold medal, University Grant commission, Bangladesh 2019<br>ii) Vice Chancellor Award 2019 for outstanding contribution in research, Shahjalal University of Science and Technology, Bangladesh<br>iii) Poster Award, BSBMB conference 2019, Chittagong, Bangladesh.<br>iv) Poster Award, Mycotoxin Workshop 2017, Bydgoszcz, Poland<br>v) Travel Bursary Award, Eurotox congress 2015, Porto, Portugal<br>vi) DAAD Scholarship 2012 for Ph.D. studies in Germany.<br>vi) Undergradte Merit Schorlarship 2005, Rajshahi University, Bangladesh
**Membership**	1) National Young Academy of Bangladesh (08/2020- to date)<br>2) Graduate Biochemistry Association in Bangladesh (02/2020-to date)

**Editorial Board Membership**<br>1/2017-present	Editorial Board Member for the journal of<br>i) Scientific Reports ii) PLOS ONE iii) Frontiers in Nutrition<br>iv) BMC Pharmacology and Toxicology and v) BMC Nutrition vi) Advances in Therapy
**Supervising and Mentoring**	i) 15 Master's level thesis students ii) 4 Undergraduate level internship students
**Language Proficiency**	Mother tongue(s): Bangla<br>Other language: English (C1, European format), German (B2, European format)
**Research Interest**	Toxicology, and Environmental Health Sciences, Human Nutrition, Public Health and Epidemiology, Biomarkers of Diseases, Cancers, Endocrinology
**Research Techniques and skills**	PCR, HPLC-FD, LC-MS/MS, ELISA, Cell Culture, Clinical markers analysis using Biochemistry analyzer, Animal experiments, Cohort and Cross-sectional study design, Epidemiological data collection and analysis, Bio-statistics SPSS and Review writing
**Teaching experience**	Experienced in teaching (>8 years) in Biochemistry and Molecular Biology at undergraduate and graduate levels.
**Research Grant**	1. Project title: Prevalence of hypertension and its associated risk factors in human population- A large scale cross-sectional study in Bangladesh<br>Position: Principal Investigator<br>Funder: Ministry and Science and Technology and SUST Research Centre Grant 2017-2018<br>2. Project title: Association of circulating biomarkers with type-2 diabetes and cardiovascular diseases: A population-based study in Bangladesh<br>Position: Principal Investigator<br>Funder: SUST Research Centre Grant 2018-2019<br>3. Project title: Association between serum xanthine oxidase activity and incidence of type 2 diabetes: a case-sectional study in Bangladeshi adults<br>Position: Co-Investigator<br>Funder: SUST Research Centre Grant 2019-2020<br>4. The relationship between serum xanthine oxidase activity and hypertension prevalence: a cross-sectional study in Bangladesh<br>Position: Principal-Investigator<br>Funder: SUST Research Centre Grant 2020-2021<br>5. Project title: Assessment of the relationship between serum liver enzymes activity and metabolic syndrome: a study on Bangladeshi adults<br>Position: Principal-Investigator<br>Funder: SUST Research Centre Grant 2021-2022<br>6. Project title: Association between serum xanthine oxidase activity and hypertension prevalence: a study on Bangladeshi adults<br>Position: Principal-Investigator<br>Funder: University Grant Commission 2020-2021 (could not carry out due stay abroad for postdoctoral studies)
**Conferences and Seminars**	***Oral presentation***<br>▪ World mycotoxin forum-meets Asia, Bangkok, 13-15.012020 (invited speaker).<br>▪ International Conference on Recent Advances in Chemistry, 7-8 February 2020, Jagannath University, Dhaka, Bangladesh<br>▪ Mycotoxin workshop. 16-18 June 2014, Goettingen, Germany<br>▪ Toxicology Seminar, 9 December 2013, IfADo, Germany<br>▪ Toxicology Seminar, 30 June 2014, IfADo, Germany<br>▪ Symposium on arsenic toxicity and bioremediation, Bangladesh Perspectives, November 11, 2010, University of Rajshahi, Bangladesh (invited speaker)<br>***Poster presentation***<br>▪ Eurotox, 13-16 September, 2015, Porto, Portugal<br>▪ Mycotoxin workshop. 1-3 June, 2015, Bratislava, Slovakia<br>▪ German Toxicology Society (DGPT). 10-12 March 2015, Kiel, Germany

	▪ Mycotoxin workshop. 16-18 June 2014, Goettingen, Germany<br>▪ German Toxicology Society (DGPT). 1-3 April, Hannover, Germany
**Publications**	83 publications (65 as the leading/corresponding author) in reputed journals<br>Research Gate: https://www.researchgate.net/profile/Nurshad_Ali<br>PubMed: https://www.ncbi.nlm.nih.gov/pubmed/?term=nurshad+ali<br>Scopus ID: https://www.scopus.com/authid/detail.uri?authorId=36236867100<br>Google Scholar: https://scholar.google.com/citations?user=yRDge5AAAAAJ&hl=en<br>**H-index 41, Total citations: 5980**

List of Publications (n=82) in peer reviewed journals

1. **Ali N**, Katsouli J, Auyang E, Bernardino de la Serna J. Microplastic and nanoplastic pollution and associated potential disease risks. Lancet Planet Health. 2025;9(12):101390. doi: 10.1016/j.lanplh.2025.101390.
2. **Ali N**. Serum Uric Acid as a Mediator of Insulin Resistance: Molecular Mechanisms and Metabolic Pathways. Endocrinol Diabetes Metab. 2026 ;9(1):e70163. doi: 10.1002/edm2.70163.
3. Mahmud F, Koley NJ, Fariha KA, **Ali N**. Serum uric acid and its association with general and abdominal obesity and elevated blood pressure among young adults in Sylhet division, Bangladesh. BMC Endocr Disord. 2026 Jan 27. doi: 10.1186/s12902-026-02175-4.
4. **Ali N**. Interactions between serum uric acid and gut microbiota: implications for metabolic health. Metabol Open. 2025 Dec 22;29:100438. doi: 10.1016/j.metop.2025.100438.
5. Tuba HR, Rana S, Fariha KA, Degen GH, **Ali N**. Aflatoxin M_1 Levels in Milk and Urine Samples of Nursing Mothers in Bangladesh: Exposure Assessment of Infants. Toxins (Basel). 2025 Aug 8;17(8):399. doi: 10.3390/toxins17080399.
6. **Ali N**, Taher A, Mahmood S. Prevalence and associated factors of co-occurring diabetes and hypertension among university academic staff and students in Bangladesh. Sci Rep. 2025;15(1):43467. doi: 10.1038/s41598-025-29332-4.
7. Mou AD, **Ali N.** Investigating the prevalence and associated factors of elevated liver enzymes and dyslipidemia during pregnancy. Sci Rep. 2025;15(1):3967. doi: 10.1038/s41598-025-88798-4.
8. Taher A, Trisha AD, Ahmed S, Begum J, Sinha F, Sarna NZ, **Ali N**. Investigating the Relationship Between Serum Uric Acid and Dyslipidemia in Young Adults in Bangladesh. Endocrinol Diabetes Metab. 2025 May;8(3):e70063. doi: 10.1002/edm2.70063.
9. **Ali N**, Mohanto NC, Newaj A, Begum J, Islam F. Evaluation of the Relationship Between Serum Uric Acid and Cardiovascular Disease: A Cross-Sectional Study in Bangladesh. Endocrinol Diabetes Metab. 2025 May;8(3):e70055. doi: 10.1002/edm2.70055.
10. **Ali N**, Taher A, Islam N, Sarna NZ, Islam F. Evaluation of the relationship between xanthine oxidase activity and metabolic syndrome in a population group in Bangladesh. Sci Rep. 2024;14(1):20380. doi: 10.1038/s41598-024-71733-4.
11. **Ali N**, Taher A, Trisha AD, Koley NJ, Fariha KA, Islam F. Prevalence of Metabolic Syndrome and its Related Factors in Bangladeshi Adults: A Cross-Sectional Study. Endocrinol Diabetes Metab. 2024;7(4):e00491. doi: 10.1002/edm2.491.
12. Hasan A, Newaj A, Trisha AD, Hafsa JM, Mohanto NC, **Ali N**. Assessment of the Relationship Between Liver Enzymes and Cardiovascular Disease: A Study in Bangladeshi Adults. Endocrinol Diabetes Metab. 2024;7(2):e00481. doi: 10.1002/edm2.481.
13. **Ali N**, Katsouli J, Marczylo EL, Gant TW, Wright S, Bernardino de la Serna J. The potential impacts of micro-and-nano plastics on various organ systems in humans. EBioMedicine. 2024;99:104901. doi: 10.1016/j.ebiom.2023.104901.
14. Das Trisha A, Hafsa JM, Hasan A, Habib A, Tuba HR, Degen GH, **Ali N**. Occurrence of ochratoxin A in breast milk and urine samples of nursing mothers in Bangladesh. Mycotoxin Res. 2024;40(1):135-146. doi: 10.1007/s12550-023-00510-5.
15. **Ali N**, Habib A, Mahmud F, Tuba HR, Degen GH. Aflatoxin M_1 Analysis in Urine of Mill Workers in Bangladesh: A Pilot Study. Toxins (Basel). 2024;16(1):45. doi: 10.3390/toxins16010045.
16. **Ali N**, Debernardi S, Kurotova E, Tajbakhsh J, Gupta NK, Pandol SJ, Wilson P, Pereira SP, Greenhalf B, Blyuss O, Crnogorac-Jurcevic T. Evaluation of urinary C-reactive protein as an early detection biomarker

for pancreatic ductal adenocarcinoma. Front Oncol. 2024 Sep 6;14:1450326. doi: 10.3389/fonc.2024.1450326.

17. **Ali N**, Xavier J, Engur M, Pv M, Bernardino de la Serna J. The impact of e-cigarette exposure on different organ systems: A review of recent evidence and future perspectives. J Hazard Mater. 2023;457:131828. doi: 10.1016/j.jhazmat.2023.131828.
18. **Ali N**. The recent burden of dengue infection in Bangladesh: A serious public health issue. J Infect Public Health. 2024;17(2):226-228. doi: 10.1016/j.jiph.2023.12.003.
19. **Ali N**, Samadder M, Shourove JH, Taher A, Islam F. Prevalence and factors associated with metabolic syndrome in university students and academic staff in Bangladesh. Sci Rep. 2023;13(1):19912. doi: 10.1038/s41598-023-46943-x.
20. **Ali N**, Samadder M, Mahmud F, Islam F. Association between liver enzymes and metabolic syndrome: a study in Bangladeshi adults. Expert Rev Endocrinol Metab. 2023;18(6):541-547. doi: 10.1080/17446651.2023.2272867.
21. **Ali N**, Kathak RR, Fariha KA, Taher A, Islam F. Prevalence of dyslipidemia and its associated factors among university academic staff and students in Bangladesh. BMC Cardiovasc Disord. 2023;23(1):366. doi: 10.1186/s12872-023-03399-1.
22. **Ali N**, Ahmed S, Mahmood S, Trisha AD, Mahmud F. The prevalence and factors associated with obesity and hypertension in university academic staff: a cross-sectional study in Bangladesh. Sci Rep. 2023;13(1):7309. doi: 10.1038/s41598-023-34574-1.
23. Kyei NNA, Waid JL, **Ali N**, Cramer B, Humpf HU, Gabrysch S. Maternal exposure to multiple mycotoxins and adverse pregnancy outcomes: a prospective cohort study in rural Bangladesh. Arch Toxicol. 2023;97(6):1795-1812. doi: 10.1007/s00204-023-03491-7.
24. Barman Z, Hasan M, Miah R, Mou AD, Hafsa JM, Trisha AD, Mahmud F, **Ali N**. Association between hyperuricemia and chronic kidney disease: a cross-sectional study in Bangladeshi adults. BMC Endocr Disord. 2023;23(1):45. doi: 10.1186/s12902-023-01304-7.
25. **Ali N**, Samadder M, Kathak RR, Islam F. Prevalence and factors associated with dyslipidemia in Bangladeshi adults. PLoS One. 2023;18(1):e0280672. doi: 10.1371/journal.pone.0280672.
26. Miah R, Fariha KA, Sony SA, Ahmed S, Hasan M, Mou AD, Barman Z, Hasan A, Mohanto NC, **Ali N**. Association of serum xanthine oxidase levels with hypertension: a study on Bangladeshi adults. Sci Rep. 2022;12(1):21727. doi: 10.1038/s41598-022-26341-5.
27. Hasan M, Fariha KA, Barman Z, Mou AD, Miah R, Habib A, Tuba HR, **Ali N**. Assessment of the relationship between serum xanthine oxidase levels and type 2 diabetes: a cross-sectional study. Sci Rep. 2022 Dec 2;12(1):20816. doi: 10.1038/s41598-022-25413-w.
28. **Ali N**, Mohanto NC, Nurunnabi SM, Haque T, Islam F. Prevalence and risk factors of general and abdominal obesity and hypertension in rural and urban residents in Bangladesh: a cross-sectional study. BMC Public Health. 2022;22(1):1707. doi: 10.1186/s12889-022-14087-8.
29. **Ali N**, Mahmud F, Akter SA, Islam S, Sumon AH, Barman DN, Islam F. The prevalence of general obesity, abdominal obesity, and hypertension and its related risk factors among young adult students in Bangladesh. J Clin Hypertens (Greenwich). 2022;24(10):1339-1349. doi: 10.1111/jch.14560.
30. Kathak RR, Sumon AH, Molla NH, Hasan M, Miah R, Tuba HR, Habib A, **Ali N**. The association between elevated lipid profile and liver enzymes: a study on Bangladeshi adults. Scientific Reports. 2022; 12(1):1711. doi: 10.1038/s41598-022-05766-y..
31. Kyei NNA, Cramer B, Humpf HU, Degen GH, **Ali N**, Gabrysch S. Assessment of multiple mycotoxin exposure and its association with food consumption: a human biomonitoring study in a pregnant cohort in rural Bangladesh. Archives of Toxicology. 2022; 96(7):2123-2138. doi: 10.1007/s00204-022-03288-0.
32. Mou AD, Barman Z, Hasan M, Miah R, Hafsa JM, Das Trisha A, **Ali N.** Prevalence of preeclampsia and the associated risk factors among pregnant women in Bangladesh. Scientific Reports. 2021; 11(1):21339. doi: 10.1038/s41598-021-00839-w.
33. Kyei NNA, Waid JL, **Ali N**, Gabrysch S. Awareness, Experience, and Knowledge of Farming Households in Rural Bangladesh Regarding Mold Contamination of Food Crops: A Cross-Sectional Study. Int J Environ Res Public Health. 2021; 18(19):10335. doi: 10.3390/ijerph181910335.

34. Molla NH, Kathak RR, Sumon AH, Barman Z, Mou AD, Hasan A, Mahmud F, Fariha KA, **Ali N**. Assessment of the relationship between serum uric acid levels and liver enzymes activity in Bangladeshi adults. Scientific Reports. 2021; 11(1):20114. doi: 10.1038/s41598-021-99623-z.
35. **Ali N**, Sumon AH, Fariha KA, Asaduzzaman M, Kathak RR, Molla NH, Mou AD, Barman Z, Hasan M, Miah R, Islam F. Assessment of the relationship of serum liver enzymes activity with general and abdominal obesity in an urban Bangladeshi population. Scientific Reports 2021, 11(1):6640. doi: 10.1038/s41598-021-86216-z.
36. Sumon AH, Islam F, Mohanto NC, Kathak RR, Molla NH, Rana S, Degen GH, **Ali N**. The Presence of Aflatoxin M_1 in Milk and Milk Products in Bangladesh. Toxins 2021; 13(7):440. https://doi.org/10.3390/toxins13070440
37. **Ali N**, Fariha KA, Islam F, Mohanto NC, Ahmad I, Hosen MJ, Ahmed S. Assessment of the role of zinc in the prevention of COVID-19 infections and mortality: A retrospective study in the Asian and European population. Journal of Medical Virology. 2021. doi: 10.1002/jmv.26932.
38. Islam F, Das Trisha A, Hafsa JM, Hasan A, Degen GH, **Ali N**. Occurrence of aflatoxin M_1 in human breast milk in Bangladesh. Mycotoxin Research 2021. 37(3):241-248. doi: 10.1007/s12550-021-00436-w.
39. **Ali N**, Fariha KA, Islam F, Mishu MA, Mohanto NC, Hosen MJ, Hossain K. Exposure to air pollution and COVID-19 severity: a review of current insights, management and challenges. Integrated Environmental Assessment and Management 2021. 10.1002/ieam.4435. doi: 10.1002/ieam.4435.
40. **Ali N**, Miah R, Hasan M, Barman Z, Mou AD, Hafsa JM, Trisha AD, Hasan A, Islam F. Association between serum uric acid and metabolic syndrome: a cross-sectional study in Bangladeshi adults. Scientific Reports 2020; 10(1):7841. doi: 10.1038/s41598-020-64884-7.
41. **Ali N**, Manirujjaman M, Rana S, Degen GH. Determination of aflatoxin M_1 and deoxynivalenol biomarkers in infants and children urines from Bangladesh. Archives of Toxicology, 2020; 94(11):3775-3786. doi: 10.1007/s00204-020-02857-5.
42. Saadi MM, Roy MN, Haque R, Tania FA Mahmood S, **Ali N**. Association of microalbuminuria with metabolic syndrome: a cross-sectional study in Bangladesh. BMC Endocrine Disorders 2020; 20(1):153. doi: 10.1186/s12902-020-00634-0
43. **Ali N.** Role of vitamin D in preventing of COVID-19 infection, progression and severity. Journal of infection and Public Health 2020; 13(10):1373-1380. doi.org/10.1016/j.jiph.2020.06.021
44. **Ali N,** Degen GH**.** Biological monitoring for ochratoxin A and citrinin and their metabolites in urine samples of infants and children in Bangladesh. Mycotoxin Research 2020; 36(4):409-417 doi: 10.1007/s12550-020-00407-7.
45. **Ali N**, Islam F. The effects of air pollution on COVID-19 infection and mortality-a review on recent evidence. Frontiers in Public Health 2020; 8:580057. doi: 10.3389/fpubh.2020.580057.
46. **Ali N**, Degen GH. Biomonitoring of zearalenone and its main metabolites in urines of Bangladeshi adults. Food and Chemical Toxicology. 2019; 130:276-283. doi:10.1016/j.fct.2019.05.036.
47. **Ali N.** Relationship between COVID-19 infection and liver injury: a review of recent data. Frontiers in Medicine 2020; 7:458. doi: 10.3389/fmed.2020.00458.
48. **Ali N**, Degen GH. Urinary biomarkers of exposure to the mycoestrogen zearalenone and its modified forms in German adults. Archives of Toxicology.2018: 92(8):2691-2700.doi:10.1007/s00204-018-2261-5
49. Anwar S, Araf Y, Khan AN, Ullah MA, Haque N, Sarkar B, Reshad R, Islam R, **Ali N**, Hosen MJ. Women's knowledge, attitude and perceptions towards COVID-19 in lower-middle-income countries: a representative cross-sectional study in Bangladesh. Frontiers in Public Health 2020; 8:571689. doi: 10.3389/fpubh.2020.571689.
50. **Ali N.** Elevated level of C-reactive protein may be an early marker to predict risk for severity of COVID-19. Journal of Medical Virology 2020; 30:10.1002/jmv.26097. doi: 10.1002/jmv.26097.
51. **Ali N.** Kidney injury in COVID-19: an emerging concern to the clinician. SN Comprehensive Clinical Medicine 2020; 24:1-2. doi: 10.1007/s42399-020-00482-y.
52. Rahman S, Islam S, Haque T, Kathak RR, **Ali N**. Association between serum liver enzymes and hypertension: a cross-sectional study in Bangladeshi adults. BMC Cardiovascular Disorders 2020; 20(1):128. doi: 10.1186/s12872-020-01411-6.

53. Islam S, Rahman S, Haque T, Sumon AH,Ahmed AZ, **Ali N.** Prevalence of elevated liver enzymes and its association with type 2 diabetes: A cross-sectional study in Bangladeshi adults. Endocrinology Diabetes and Metabolism 2020; 3(2):e00116. doi:10.1002/edm2.116.
54. **Ali N**. Is SARS-CoV-2 associated with liver dysfunction in COVID-19 patients? Clinics and Research in Heapatology and Gastroenterology 2020; 44(4):e84-e86. doi.org/10.1016/j.clinre.2020.05.002.
55. **Ali N**, Hossain K. Liver injury in severe COVID-19 infection: current insights and challenges. Expert Review of Gastroenterology & Hepatology 2020, 44(4):e84-e86. doi:10.1080/17474124.2020.1794812.
56. Haque T, Rahman S, Islam S, Molla NH, **Ali N.** Assessment of the relationship between serum uric acid and glucose levels in healthy, prediabetic and diabetic individuals. Diabetology and Metabolic Syndrome 2019: 11:49. doi: 10.1186/s13098-019-0446-6.
57. **Ali N**, Mahmood S, Islam F, Rahman S, Haque T, Islam S, Rahman S, Haque N, Mosaib G, Perveen R, Khanum FA. Relationship between serum uric acid and hypertension:a cross-sectional study in Bangladeshi adults. Scientific Reports. 2019; 9:9061. doi:10.1038/s41598-019-45680-4.
58. **Ali N**. Aflatoxins in rice: Worldwide occurrence and public health perspectives. Toxicology Reports 2019; 6:1188-1197. doi: 10.1016/j.toxrep.2019.11.007.
59. **Ali N**, Degen GH. Citrinin biomarkers: a review of recent data and application to human exposure assessment. Archives of Toxicology. 2019; 93(11):3057-3066. doi: 10.1007/s00204-019-02570-y.
60. Malir F, Louda M, Ostry V, Toman J, **Ali N**, Grosse Y, Malirova E, Pacovsky J, Pickova D, Brodak M, Pfohl-Leszkowicz A, Degen GH. Analyses of biomarkers of exposure to nephrotoxic mycotoxins in a cohort of patients with renal tumours. Mycotoxin Research. 2019; 35(4):391-403. doi: 10.1007/s12550-019-00365-9.
61. **Ali N,** Rahman S, Islam S, Haque T, Molla NH, Sumon AH, Kathak RR, Asaduzzaman M, Islam F, Mohanto NC, Hasnat MA, Nurunnabi SM, Ahmed S. The relationship between serum uric acid and lipid profile in Bangladeshi adults. BMC Cardiovascular Disorders. 2019, 19(1):42. doi: 10.1186/s12872-019-1026-2.
62. **Ali N**, Perveen R, Rahman S, Mahmood S, Rahman S, Islam S, Haque T, Sumon AH, Kathak RR, Molla NH, Islam F, Mohanto NC, Nurunnabi SM, Ahmed S, Rahman M. Prevalence of hyperuricemia and the relationship between serum uric acid and obesity: A study on Bangladeshi adults. PLoS One. 2018; 13(11):e0206850. doi: 10.1371/journal.pone.0206850.
63. **Ali N**, Hossain K, Degen GH. Blood plasma biomarkers of citrinin and ochratoxin A exposure in young adults in Bangladesh. Mycotoxin Research. 2018; 34(1):59-67. doi: 10.1007/s12550-017-0299-5.
64. **Ali N**, Mahmood S, Manirujjaman M, Perveen R, Nahid AA, Ahmed S, Khanum FA, Rahman M. Hypertension prevalence and influence of basal metabolic rate on blood pressure among adult students in Bangladesh. BMC Public Health. 2017, 18(1):58. doi: 10.1186/s12889-017-4617-9.
65. Degen GH, **Ali N**, Ursula Gundert-Remy U. Preliminary data on citrinin kinetics in humans and their use to estimate citrinin exposure based on biomarkers. Toxicology Letters 2017; pii: S0378-4274(17)31418-2. doi: 10.1016/j.toxlet.2017.
66. **Ali N**. Co-occurrence of citrinin and ochratoxin A in rice in Asia and its implications for human health. Journal of the Science of Food and Agriculture. 2017; 98(6):2055-2059. doi: 10.1002/jsfa.8667.
67. **Ali N**, Blaszkewicz M, Degen GH. Determination of aflatoxin M_1 in urines indicates frequent dietary exposure to aflatoxin B_1 in the Bangladeshi population. International Journal of Hygiene and Environmental Health 2017; 220:271-281. doi: 10.1016/j.ijheh.2016.11.002.
68. **Ali N**, Muñoz K, Degen GH. Ochratoxin A and its metabolites in urines of German adults-An assessment of variables in biomarker analysis. Toxicology Letters 2017; 275:19-26. doi: 10.1016/j.toxlet.2017.04.013.
69. Mahmood S, Rahman M, Biswas SK, Saqueeb SN, Zaman S, Manirujjaman M, Perveen R, **Ali N.** Vitamin D and Parathyroid Hormone Status in Female Garment Workers: A Case-Control Study in Bangladesh. Biomed Research International. 2017; 2017:4105375. doi: 10.1155/2017/4105375.
70. **Ali N**, Blaszkewicz M, Alim A, Hossain K, Degen GH. Urinary biomarkers of ochratoxin A and citrinin exposure in two Bangladeshi cohorts: follow-up study on regional and seasonal influences. Archives of Toxicology 2016, 90:2683-2697. doi:10.1007/s00204-015-1654-y.
71. **Ali N**, Blaszkewicz M, Degen GH. Assessment of deoxynivalenol exposure among Bangladesh and German adults by a biomarker based approach. Toxicology Letters 2016; 258: 20–28.

72. **Ali N**, Blaszkewicz M, Manirujjaman M, Degen GH. Biomonitoring of concurrent exposure to ochratoxin A and citrinin in pregnant women in Bangladesh. Mycotoxin Research 2016; 32:163-72. Doi: 10.1007/s12550-016-0251-0.
73. **Ali N,** Hossain K, Blaszkewicz M, Rahman M Mohanto NC, Alim A, Degen GH. Occurrence of aflatoxin M1 in urine from rural and urban adult cohorts in Bangladesh. Archives of Toxicology 2016; 90:1749-55. doi: 10.1007/s00204-015-1601-y.
74. Föllmann W, **Ali N**, Blaszkewicz M, Degen GH. Biomonitoring of mycotoxins in urine: Pilot study in mill workers**.** Journal of Toxicology and Environmental Health, Part A 2016, 79:1015-1025.
75. **Ali N**, Blaszkewicz M, Nahid AA, Rahman M, Degen GH. Deoxynivalenol exposure assessment for pregnant women in Bangladesh. Toxins (Basel) 2015; 7:3845–57
76. **Ali N**, Blaszkewicz M, Degen GH. Occurrence of the mycotoxin citrinin and its metabolite dihydrocitrinone in urines of German adults. Archives of Toxicology 2015; 89:573–8.
77. Gerding J, **Ali N**, Schwartzbord J, Cramer B, Brown DL, Degen GH, Humpf HU. A comparative study of the human urinary mycotoxin excretion patterns in Bangladesh, Germany and Haiti using a rapid and sensitive LC-MS/MS approach. Mycotoxin Research 2015; 31:127–36.
78. **Ali N,** Blaszkewicz M, Mohanto NC, Rahman M, Alim A, Hossain K, Degen GH. First results on citrinin biomarkers in urines from rural and urban cohorts in Bangladesh. Mycotoxin Research 2015; 31: 9-16. (IF 4.08).
79. **Ali N**, Blaszkewicz M, Manirujjaman M, Perveen R, Nahid AA, Mahmood S, Rahman M, Hossain K, Degen GH. Biomonitoring of ochratoxin A in blood plasma and exposure assessment of adult students in Bangladesh. Molecular Nutrition and Food Research 2014; 58: 2219–2225.
80. Huda N ,Hossain S, Rahman M, Karim MR, Islam K, Mamun AA, Hossain MI, Mohanto NC, Alam S, Aktar S, Arefin A, **Ali N**, Salam KA, Aziz A,Saud ZA, Miyataka H, Himeno S, Hossain K. Elevated levels of plasma uric acid and its relation to hypertension in arsenic-endemic human individuals in Bangladesh. Toxicology and Applied Pharmacology 2014; 281: 11–18.
81. Islam K, Haque A, Karim MR, Fajol A, Hossain E, Salam KA, **Ali N**, Saud ZA, Rahman M, Rahman M, Karim MR, Sultana P, Hossain M, Akhand AA, Miyataka H, Himeno S, Hossain K. Dose-response relationship between arsenic exposure and the serum enzymes for liver function tests in the individuals exposed to arsenic: a cross sectional study in Bangladesh. Environmental Health 2011; 10:64.
82. Karim MR, Haque A, Islam K, **Ali N,** Salam KA, Saud ZA, Hossain E, Fajol A, Akhand AA, Himeno S, Hossain K. Protective effects of the dietary supplementation of turmeric (*Curcuma longa L.)* on sodium arsenite-induced biochemical perturbation in mice. Bangladesh Medical Research Council Bulletin 2010; 36:82–88.
83. Karim MR, Salam KA, Hossain E, Islam K, **Ali N,** Haque A, Saud ZA, Yeasmin T, Hossain M, Miyataka H, Himeno S, Hossain K. Interaction between chronic arsenic exposure via drinking water and plasma lactate dehydrogenase activity. Science of the Total Environment 2010; 409: 278–83
84. **Ali N,** Hoque MA, Haque A, Salam KA, Karim MR, Rahman A, Islam K, Saud ZA, Khalek MA, Akhand AA, Hossain M, Mandal A, Karim MR, Miyataka H, Himeno S, Hossain K. Association between arsenic exposure and plasma cholinesterase activity: a population based study in Bangladesh. Environmental Health 2010; 9:36.
